# Co-infections of Adenovirus Species in Previously Vaccinated Patients

**DOI:** 10.3201/eid1206.050245

**Published:** 2006-06

**Authors:** Gary J. Vora, Baochuan Lin, Kevin Gratwick, Carolyn Meador, Christian Hansen, Clark Tibbetts, David A. Stenger, Marina Irvine, Donald Seto, Anjan Purkayastha, Nikki E. Freed, Marylou G. Gibson, Kevin Russell, David Metzgar

**Affiliations:** * Naval Research Laboratory, Washington, DC, USA;; † Epidemic Outbreak Surveillance Consortium, Falls Church, Virginia, USA;; ‡ Naval Health Research Center, San Diego, California, USA;; § Nova Research Inc., Alexandria, Virginia, USA;; ¶ George Mason University, Manassas, Virginia, USA;; # Virapur, LLC, San Diego, California, USA

**Keywords:** Adenovirus, coinfection, microarray, respiratory infection, molecular diagnostics, research

## Abstract

Despite the success of the adenovirus vaccine administered to US military trainees, acute respiratory disease (ARD) surveillance still detected breakthrough infections (respiratory illnesses associated with the adenovirus serotypes specifically targeted by the vaccine). To explore the role of adenoviral co-infection (simultaneous infection by multiple pathogenic adenovirus species) in breakthrough disease, we examined specimens from patients with ARD by using 3 methods to detect multiple adenoviral species: a DNA microarray, a polymerase chain reaction­ (PCR)–enzyme-linked immunosorbent assay, and a multiplex PCR assay. Analysis of 52 samples (21 vaccinated, 31 unvaccinated) collected from 1996 to 2000 showed that all vaccinated samples had co-infections. Most of these co-infections were community-acquired serotypes of species B1 and E. Unvaccinated samples primarily contained only 1 species (species E) associated with adult respiratory illness. This study highlights the rarely reported phenomenon of adenoviral co-infections in a clinically relevant environment suitable for the generation of new recombinational variants.

## Abstract 2

Adenoviral infections associated with respiratory illness in military trainees involve multiple co-infecting species and serotypes.

Adenoviruses cause an estimated 8% of clinically relevant viral disease globally (1). Human adenoviruses (HAdVs) are divided into 51 serotypes (HAdV-1–HAdV-51) on the basis of type-specific antiserum-mediated neutralization of infectivity (determined primarily by the hexon coat protein and terminal knob portion of the fiber protein) (2) and into 6 species, also referred to as subgenera or subgroups (HAdV-A, B, C, D, E, and F) on the basis of hemagglutination inhibition and biochemical criteria (3–5). Species HAdV-B is further classified into subspecies B1 and B2 (3). In civilian populations, HAdV-B1 serotypes 3, 7, 16, and 21; HAdV-E serotype 4; and 1 member of subspecies HAdV-B2, serotype 14, cause outbreaks of illness ranging from mild febrile respiratory infections and conjunctivitis to potentially lethal disseminated infections in both adults and children (1,6). HAdV-C serotypes 1, 2, 5, and 6 cause locally endemic upper respiratory infections in infants and children (7,8) and occasional outbreaks in adults. Other HAdV species are usually not associated with respiratory disease in otherwise healthy humans.

HAdV seems to have found a particularly destructive niche in military training camps. HAdV-B1 serotypes 3, 7, and 21; HAdV-E serotype 4; and HAdV-B2 serotype 14 have caused severe outbreaks of acute respiratory disease (ARD) among military recruits in training centers (9,10). Before initiation of an HAdV vaccination program in 1971, outbreaks occurred regularly, and ≈1 of 6 recruits in affected camps required hospitalization (1). Systematic vaccination of recruits against the 2 most common agents of ARD in the military, HAdV serotypes 4 and 7, decreased HAdV-specific respiratory illness by 95% to 99% and overall respiratory illness rates by 50% to 60% (11–13). Despite this general efficacy, breakthrough infection (infection of vaccinated persons by the vaccine-targeted adenoviral serotypes) was still regularly reported (14). Production of the vaccine was suspended in 1996, at which point vaccination became sporadic until the existing stocks ran out in 1999. ARD rates quickly returned to prevaccine levels, with HAdV as the apparent causal agent. As a result, reintroduction of the vaccine is being actively pursued (15).

To explore the possibility that unique HAdV strains were causing ARD in vaccinated persons, throat swab samples were selected from the Naval Health Research Center population-based febrile respiratory illness surveillance collection from vaccinated (n = 21) and unvaccinated (n = 31) recruits who reported ARD from 1996 to 2000. Samples were chosen that had tested positive for serotypes 4 or 7 by culture and serotypic antibody neutralization. The gene coding for the primary adenoviral antigen, the hexon coat protein, was sequenced from these isolates. The sequence data suggested that the detectable serotype 4 and 7 strains apparently responsible for breakthrough infection were the same as those circulating in unvaccinated military and civilian populations (16). In this study, we reanalyze the same set of samples to identify co-infections with multiple HAdV strains and to address what role co-infections may play in breakthrough infection.

## Materials and Methods

### Sample Collection and Preparation

Samples were collected as throat swabs into viral transport medium from military recruits with ARD at a variety of training camps as previously described (16). The throat swab samples were cultured on A549 cells and tested by using standard serologic methods. Both original swabs and in vitro tissue culture fluid (ITCF) samples were stored at –80°C. Samples that initially tested positive for serotypes 4 or 7 by culture and microneutralization were chosen for analysis and grouped by previous vaccination status. DNA extracts from ITCF samples were collected and used in molecular assays. Collection details and symptom definitions were previously reported (16), and sample details are shown in Table 1 and Table 2. Initially, 13 unidentified (blinded) samples were sent by the Naval Health Research Center to the Naval Research Laboratory personnel for testing. After the initial 13 samples showed a high rate of respiratory HAdV co-infection, primarily in vaccinated persons, an additional 39 samples were tested in an unblinded fashion.

**Table 1:** Naval Health Research Center data for molecular detection of adenoviral co-infections in vaccinated and unvaccinated patients with febrile respiratory illness*

Original designation†	Vaccination date	Microneu-tralization‡	Multiplex PCR	Species-specific PCR (B, C, E)	Sequencing§	GenBank accession no.
7151.AV5.V.98.FJ	5 Nov 1997	4	B, C	B, C, E	5, 21	AY337237
7137.AV4.V.97.FJ	1 Dec 1997	4	E	B, E	4 variant	
7274.AV4.V.98.FJ	11 Feb 1998	4	E, B	E, B	4 vaccine (Δ = 2)	AF065062
7307.AV5.V.98.FJ	9 Feb 1998	4	C, B	B, C, E	5	
7333.AV4.V.98.FJ	25 Mar 1998	4	E	E, **B**	4 variant	AY337242
4185.AV4.V.97.FLW	24 Mar 1997	4	E	B, E	4 variant, 7h	AY337252
4476.AV4.V.97.FLW	24 Oct 1997	4	E	B, E	4 variant	AY337249
79.AV4.V.96.GL	7 Oct 1996	4	E	E	4 vaccine (Δ = 3)	AF065062
141.AV7.V.96.GL	12 Nov 1996	7	B	B	7d2 (prototype)	AY337258
275.AV4.V.97.GL	31 Jan 1997	4	E	E	4 vaccine (Δ = 3)	AY337239
1212.AV7.V.97.GL	29 Sep 1997	7	B	B, E	7d2 (Δ = 2)	AY337255
1108.AV7.V..97.GL	8 Oct 1997	7	E, B	B, E	7 vaccine (Δ = 0)	AF065067
1122.AV7.V.97.GL	8 Oct 1997	7	B	B	7d2 (Δ = 2)	AF321311
1150.AV7.V.97.GL	8 Oct 1997	7	B	B, E	7 vaccine (Δ = 2)	AY337254
1152.AV7.V.97.GL	8 Oct 1997	7	B	B	7 vaccine (Δ = 1)	AY337253
1186.AV7.V.97.GL	8 Oct 1997	7	B	B, E	7d2 (Δ = 2)	AF321311
1251.AV7.V.97.GL	8 Oct 1997	7	B	B	7d2 (Δ = 2)	AF321311
1275.AV7.V.97.GL	8 Oct 1997	7	B	B, E	7 vaccine (Δ = 1)	AY337257
1302.AV7.V.97.GL	8 Oct 1997	7	B	B, E	7 vaccine (Δ = 2)	AY337256
1649.AV7.V.98.GL	13 Jan 1998	7	B	B	7d2 (Δ = 2)	AF321311
1856.AV5.V.98.GL	25 Mar 1998	4	C	B, C, **E**	5, 7h	
60406.AV7.99.FB		7	B	B	7 vaccine (Δ = 2)	AY337256
60673.AV4.00.FB		4	E	E	4 variant	AY337237
60691.AV4.00.FB		4	E	E, **B**	4 variant	AY337238
60697.AV4.00.FB		4	E	E	4 variant	AY337246
60708.AV4.00.FB		4	E	E	4 variant	AY337237
60716.AV4.00.FB		4	E	E	4 variant	AY337247
CHPPM2.AV4.00.FB			E	E, **B**	4 variant	AY337237
CHPPM9.AV4.00.FB		4	E	E, **B**	4 variant	AY337237
CHPPM13.AV4.00.FB			E	E, **B**	4 variant	AY337237
CHPPM29.AV4.00.FB		4	E	E	4 variant	AY337237
CHPPM44.AV4.00.FB		4	E	E	4 variant	AY337237
7372.AV5.98.FJ		4	C	B, C, E	5, 7h	
40098.AV4.98.FJ		4	E	E	4 variant	AY337241
40160.AV4.98.FJ		4	E	E, **B**	4 variant	AY337237
40183.AV4.98.FJ		4	E	E	4 variant	AY337237
40781.AV4.99.FJ		4	E	E	4 variant	AY337238
40844.AV4.99.FJ		4	E	E	4 variant	AY337237
41059.AV4.99.FJ		4	E	E	4 variant	AY337237
10060.AV4.98.GL			E	E	4 variant	AY337237
10190.AV4.98.GL		4	E	E, **B**	4 variant	AY337237
10206.AV4.98.GL		4	E	E	4 variant	AY337244
10213.AV4.98.GL		4	E	E	4 variant	AY337240
10257.AV4.98.GL		4	E	E	4 variant	AY337237
10258.AV4.98.GL		4	E	E	4 variant	AY337237
10756.AV4.00.GL		4	E	E	4 variant	AY337243
50108.AV4.00.LAC			E	B, E	4 variant	AY337251
20044.AV4.98.MCRD		4	E	B, E	4 variant	AY337248
20139.AV4.98.MCRD			E	E	4 variant	AY337237
20142.AV4.98.MCRD			E	E	4 variant	AY337250
20143.AV4.98.MCRD			E	E, **B**	4 variant	AY337237
20145.AV4.98.MCRD			E	E	4 variant	AY337245

*PCR, polymerase chain reaction. Letters or numbers in **boldface** indicate weak positives. †Acquisition number, serotype, isolation year, and isolation location. ‡Results are listed as serotypes. Species B1 includes serotypes 3, 7, 16, 21; species C includes serotypes 1, 2, 5, 6; and species E includes serotype 4. §Variant/vaccine grouping based on the hexon gene sequence defined by Blasiole et al. (16). Δ = # reflects number of base substitutions from vaccine strain in 1,490 bp of the hexon sequence (16). The 7d2 designation is based on that of Blasiole et al. (16). The 7h designation based on fiber gene sequence is as defined by Kajon and Wadell (17).

**Table 2:** Naval Research Laboratory data for molecular detection of adenoviral co-infections in vaccinated and unvaccinated patients with febrile respiratory illness*

Original designation†	Vaccination date	Microarray‡	Adenovirus Consensus kit	PCR determination‡	
				Positive	Negative
7151.AV5.V.98.FJ	5 Nov 1997	C, 21	C, B1	5, 21	B2
7137.AV4.V.97.FJ	1 Dec 1997	4, C, B2	E	4, 1	
7274.AV4.V.98.FJ	11 Feb 1998	4, 21, C, B2	E, B1, B2	4, 21, B2	C
7307.AV5.V.98.FJ	9 Feb 1998	C, 21	C	C	21
7333.AV4.V.98.FJ	25 Mar 1998	4, C, B2	E	4, 1, 5, B2	
4185.AV4.V.97.FLW	24 Mar 1997	4, C, B2	E, B2, F, B1	4, B2	C
4476.AV4.V.97.FLW	24 Oct 1997	4, C, B2	E, B2, F, B1	4, 5, B2	
79.AV4.V.96.GL	7 Oct 1996	4, C, 7	E	4, C, B2	7
141.AV7.V.96.GL	12 Nov 1996	7, 4, **3**	B1, B2, E	7, 4, B2	3
275.AV4.V.97.GL	31 Jan 1997	4, C, 7	E, B2, F, B1	4, C, B2	7
1212.AV7.V.97.GL	29 Sep 1997	7, 4, **3**	B1, E, F	7, 4, 3, F	
1108.AV7.V..97.GL	8 Oct 1997	7, 4, C, **3**	B1, E, F	7, 4, C	3
1122.AV7.V.97.GL	8 Oct 1997	7, C, **3**	B1	7, C	3
1150.AV7.V.97.GL	8 Oct 1997	7, 4, **3**	B1, E, F	7, 3, F	4
1152.AV7.V.97.GL	8 Oct 1997	7, 4, **3**	B1	7, 4	3
1186.AV7.V.97.GL	8 Oct 1997	7, 4	B1, E, F	7	4
1251.AV7.V.97.GL	8 Oct 1997	7, 4, **3**	B1, E, F	7	4, 3
1275.AV7.V.97.GL	8 Oct 1997	7, 4, **3**	B1, E	7, 4, 3	
1302.AV7.V.97.GL	8 Oct 1997	7, 4, **3**	B1, E, F	7, 4	3
1649.AV7.V.98.GL	13 Jan 1998	7, 3, 4	B1	7, 3	4
1856.AV5.V.98.GL	25 Mar 1998	C, 7	C	C	7
60406.AV7.99.FB		7	B1	7	
60673.AV4.00.FB		4, C	E	4	C
60691.AV4.00.FB		4, C	E	4	C
60697.AV4.00.FB		4, C	E	4, 1	
60708.AV4.00.FB		4, C	E	4	C
60716.AV4.00.FB		4, C	E	4	C
CHPPM2.AV4.00.FB		4, C	E	4	C
CHPPM9.AV4.00.FB		4, C	E	4	C
CHPPM13.AV4.00.FB		4, C	E	4	C
CHPPM29.AV4.00.FB		4, C	E	4	C
CHPPM44.AV4.00.FB		4, C	E	4	C
7372.AV5.98.FJ		C, **7**	C	C	7
40098.AV4.98.FJ		4	E, F	4, F	
40160.AV4.98.FJ		4	E	4	
40183.AV4.98.FJ		4	E	4	
40781.AV4.99.FJ		4, C	E, B2	4, B2	C
40844.AV4.99.FJ		4, C	E, B2	4, B2	C
41059.AV4.99.FJ		4, C	E, B2, F	4, C, B2, F	
10060.AV4.98.GL		4, C, B2	E, B2	4, B2	C
10190.AV4.98.GL		4	E	4	
10206.AV4.98.GL		4, C, B2	E, B2	4, B2	C
10213.AV4.98.GL		4, C, **B2**	E, B2, F	4, B2	
10257.AV4.98.GL		4, B2	E	4, B2	
10258.AV4.98.GL		4	E	4	
10756.AV4.00.GL		4	E	4	
50108.AV4.00.LAC		4, B2	E	4, B2	
20044.AV4.98.MCRD		4, C, 7, **3**	E	4, 1, B2	7, 3
20139.AV4.98.MCRD		4	E	4	
20142.AV4.98.MCRD		4	E	4	
20143.AV4.98.MCRD		4, C, B2	E, B2, F	4, C, B2, F	
20145.AV4.98.MCRD		4, C, B2	E, B2, F	4, B2	C

*PCR, polymerase chain reaction. Species and serotype are listed in order of predominance. Letters or numbers in **boldface** indicate weak positives. †Acquisition number, serotype, isolation year, and isolation location. ‡Results are listed as serotypes or species. Species B1 includes serotypes 3, 7, 16, 21; species C includes serotypes 1, 2, 5, 6; and species E includes serotype 4.

### Microarray-based Genotyping

One microliter of purified DNA extract from each of the 52 ITCF samples was used as the template in 50-μL degenerate PCR amplifications targeting portions of the *E1A*, hexon, and fiber genes. The primers, degenerate polymerase chain reaction (PCR) amplification protocol, probes, and microarray fabrication techniques have been previously described (18). Once constructed, the spotted microarrays were blocked with a 3% bovine serum albumin–casein solution (BSA-C) for 15 min at room temperature, and the slides were outfitted with MAUI Mixer DC hybridization chambers (BioMicro Systems, Salt Lake City, UT, USA). Twenty-microliter hybridization reactions (13.6 μL biotinylated degenerate PCR amplicons, 2 μL 3% BSA-C, 4 μL 20× SSC (0.3 mol/L sodium citrate, 3.0 mol/L NaCl, pH 7.0), and 0.4 μL 10% sodium dodecyl sulfate [SDS]) were denatured for 3 min at 98°C and immediately applied to the microarrays. Hybridizations were performed for 2 h at 63°C in a MAUI Hybridization System (BioMicro Systems). Slides were then washed twice with 4× SSC-0.2% SDS buffer and 2× SSC buffer, and hybridization was detected by the sequential addition of Cy5-conjugated mouse antibiotin immunoglobulin G (IgG) (Jackson ImmunoResearch, West Grove, PA, USA) and Cy5-conjugated goat antimouse IgG (Jackson ImmunoResearch). Images were obtained with a ScanArray Lite confocal laser scanning system (Perkin-Elmer, Torrance, CA, USA) at a laser power of 60 to 80 and a photomultiplier tube gain of 60 to 80. The fluorescent signal from each microarray element was considered positive only when its quantified intensity was >3× that of known internal negative control elements. Each ITCF sample was subjected to 2 to 5 independent amplification and hybridization experiments. Hybridization patterns unique to specific serotypes were determined empirically with prototype strains (18). Although members of species HAdV-B1 often produced complex hybridization profiles (18), these profiles were unique, reproducible, and readily identifiable in both single infections and co-infections.

### Adenovirus Consensus PCR–Enzyme-linked Immunosorbent Assay

We used a commercially available kit capable of typing adenoviruses to the species level to confirm the results obtained with microarray analyses. Briefly, the Adenovirus Consensus kit (Argene, North Massapequa, NY, USA) uses a PCR–enzyme-linked immunosorbent assay that amplifies a fragment from the adenovirus virus-associated (VA) RNA gene and subsequently detects and types the amplicon with species-specific biotinylated oligonucleotide probes in a colorimetric microwell format (19). Results obtained with the kit were interpreted according to the manufacturer's adenovirus typing protocol.

### Adenovirus-specific PCR

The species-specific PCR amplification was performed with previously published primers (20) and a Multiplex PCR Kit (Qiagen, Valencia, CA, USA) according to the manufacturer's instructions (with 0.5× Q solution). These amplifications were performed in 25-μL reaction volumes at an annealing temperature of 52°C. In general, the PCRs were performed in an iCycler (Bio-Rad, Hercules, CA, USA) and analyzed by electrophoresis on 1.5% agarose gels. Monoplex PCR was performed under identical reaction conditions, except that the same primers were used in independent reactions. Sequencing reactions and microneutralization assays were performed as previously described (21,22). Serotype-specific PCR assays (Table 1 and Table 2) were verified as described (20,23–26), with occasional substitutions of polymerase type and annealing temperature adjustments.

### Co-infection Separation

Limiting dilutions of ITCF sample 7151 were plated on A549 cells and allowed to adsorb for 16 hours, after which agarose overlays (0.4% agarose in Dulbecco minimal essential medium, 2% fetal bovine serum, 4 mmol/L glutamine) were added to each infected monolayer. Well-separated virus plaques were picked 5 days postinfection, placed into viral transport medium, and tested by PCR for HAdV-B and HAdV-C. A second round of plaque purification was performed on several plaque isolates that were treated with 0.05% Triton-X 100 to potentially disrupt virus clumps before their dilution and plating. After 6 hours of adsorption, the original inoculum was removed, and the monolayers were overlayed with agarose solution. The newly formed plaques were tested as described above.

## Results

By using a new 70-mer spotted microarray (18), a PCR–enzyme-linked immunosorbent assay (19), and a species-specific multiplex PCR assay (20), we generated data profiles for each of the 52 tissue culture-amplified samples; the raw data from 2 of these samples are shown as representative examples (Figure). The microarray profile of vaccinated sample 7274 detected HAdV-4 (species E), HAdV-21 (species B1), HAdV-C, and HAdV-B2 according to previously validated hybridization patterns (18) (Figure, panel A). Except for detection of an apparent low-level HAdV-C co-infectant, the results of the Adenovirus Consensus kit (HAdV-B1, HAdV-B2, and HAdV-E) (Figure, panel B), multiplex and monoplex species-specific PCR (HAdV-B and HAdV-E) (Figure, panel C), and serotype-specific PCR (HAdV-4, HAdV-21, and HAdV-B2) (Figure, panel D) confirmed the microarray-based identification of multiple adenoviral strains in sample 7274.

**Figure:**
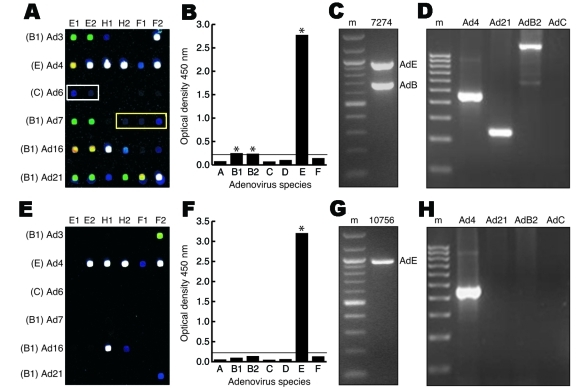
Molecular methods used to identify human adenovirus (HAdV) co-infections. A–D) Vaccinated sample 7274. A) Microarray hybridization profile. White and yellow rectangles indicate low-positive HAdV-C and HAdV-B2 targets, respectively. Spot colors denote hybridization signal intensity (white > yellow > green > blue). Species and corresponding serotype designations are indicated on the left. Probe designations (E1, E2 = serotype-specific E1A probes; H1, H2 = serotype-specific hexon probes; F1, F2 = serotype-specific fiber probes) are indicated above each array. B) Adenovirus Consensus kit optical density values. *, amplification positive. The horizontal line is the manufacturer's significance threshhold. C) Multiplex species-specific polymerase chain reaction (PCR). m, molecular mass marker. Species designations are to the right of the corresponding band. D) PCR verification with independent serotype or species-specific primers. E–H) Unvaccinated sample 10756. E) Microarray hybridization profile. F) Adenovirus Consensus kit optical density values. G) Multiplex species-specific PCR. H) PCR verification with independent serotype-specific primers.

In contrast, the microarray profile of unvaccinated sample 10756 detected a single serotype, HAdV-4 (Figure, panel E). The microarray-based finding was verified by results of the Adenovirus Consensus kit (Figure, panel F), multiplex species-specific PCR (Figure, panel G), and HAdV-4 serotype-specific PCR (Figure, panel H). The data profiles for all 52 samples assembled and compared in this manner are shown in Table 1 and Table 2. Dual, triple, and quadruple infections were found in all 21 of the vaccinated samples and in 14 of the 31 unvaccinated samples tested (Table 1 and Table 2).

Previously vaccinated persons showed a high rate of co-infection with both species commonly associated with ARD (HAdV-B1 and HAdV-E), whereas unvaccinated persons were primarily infected with HAdV-E. Since HAdV-4 and HAdV-7 are the 2 most common ARD-associated serotypes, that they were also the most commonly paired respiratory pathogenic co-infectants detected in vaccinated persons is not surprising. When the vaccine was used, the rates of other respiratory adenoviruses were much higher than when the vaccine was not used (16). However, these isolates were chosen for study because they yielded antigenic signals consistent with either HAdV-4 or HAdV-7 and were therefore expected to contain at least 1 of these 2 viruses as the highest titer adenoviral components (Table 1) (16).

The ability of the microarray to identify to the serotype level resulted in the detection of the greatest number of co-infections, despite its inability to detect members of species B2 when a co-infecting HAdV-7 was present (hybridization pattern interference) and members of species F that were not targeted (Table 3). Microarray-based identification of multiple ARD-associated serotypes from diverse HAdV-B1 species (serotypes 3, 7, and 21) was necessary because co-infections with these serotypes would not have been indicated or resolved by methods limited to species-level identification.

**Table 3:** Human adenovirus load detected with molecular identification methods*

Method	Status	No. samples with X co-infectant strains			
		X = 1	X = 2	X = 3	X = 4
Microarray	Vaccinated	0	4	15	2
	Unvaccinated	9	16	5	1
Adenovirus Consensus kit	Vaccinated	8	2	8	3
	Unvaccinated	22	5	4	0
Multiplex PCR	Vaccinated	17	4	0	0
	Unvaccinated	31	0	0	0
Monoplex PCR†	Vaccinated	7	11	3	0
	Unvaccinated	21	9	1	0

*PCR, polymerase chain reaction. †Species-specific PCR from Table 1, Naval Health Research Center data.

Although most apparent co-infections could be verified by each of the primary methods tested and by serotype-specific PCR (e.g., single infections: 10756, 60406, 20142; co-infections: 1212, 7151, 7274), some could not be verified (e.g., 60691, CHPPM2). Those co-infectant signals that could not be verified were usually weak positives. The strains responsible for these signals appeared to be subordinate co-infectants because the predominant serotype or species signals generated for the associated samples by the microarray, Adenovirus Consensus kit, and serotype-specific PCR were corroborated in every case and matched the results obtained from the sequencing experiments previously reported (16).

The microarray and Adenovirus Consensus kit use detection and signal amplification techniques that enhance assay sensitivity and thus render them more sensitive than traditional PCR/agarose gel visualization techniques, as shown by the number of triple and quadruple co-infections detected with these techniques (Table 3). Thus, attempting to corroborate these methods with the 3 PCR-based methods used was not completely successful. Nevertheless, most of the positive results from these tests were verified by comparing the microarray and Adenovirus Consensus kit results or by comparison with the results from independent methods such as microneutralization, hexon sequence analysis, serotype-specific PCR that uses primers not used in the multiplex tests, and PCR amplicon sequencing (Table 1, Table 2, and Table 4). These results suggest that these methods can identify and corroborate HAdV co-infections and that, in general, the HAdV load in ARD patients is more complex than previously thought.

**Table 4:** Human adenovirus (HAdV) species and serotype-specific primers

Name	Sequence	Target gene	Reference
Primer 1	CTT GGT CTA CGA CCA GAC GG		
Primer 3	GTT TGC TCA TGA ACA TGG CCA GAT CGC AC	Species B2 E3	(26)
F30	CTT CAA CCC TGT CTA CCC TAT GAA		
F969	TTC TCT AAT GTA GTA AAA GG	HAdV11 fiber	(25)
HsgF1	ATT TCT ATT CCT TCG CG		
HsgF2	TCA GGC TTG GTA CGG CC	Species F hexon	(24)
HsgC1	ACC TTT GAC TCT TCT GT		
HsgC2	TCC TTG TAT TTA GTA TC	Species C hexon	(24)
Ad3F	GGT AGA GAT GCT GTT GCA GGA		
Ad3R	CCC ATC CAT TAG TGT CAT CGG T	HAdV3 hexon	(23)
Ad7F	GGA AAG ACA TTA CTG CAG ACA		
Ad7R	AAT TTC AGG CGA AAA AGC GTC A	HAdV7 hexon	(23)
Ad21F	GAA ATT ACA GAC GGC GAA GCC		
Ad21R	AAC CTG CTG GTT TTG CGG TTG	HAdV21 hexon	(23)
Ad4F5	GTT GCT AAC TAC GAT CCA GAT ATT G		
Ad4R4	CCT GGT AAG TGT CTG TCA ATC C	HAdV4 hexon	This study
Ad7F-F	ACA ACT GCC TAT CCT TTC AAT G		
Ad7F-R	GAC CAA GTT ACA CGA ATA CAA TAT G	HAdV7 fiber	This study
Ad5 E1A-F1	CCT AAA ATG GCG CCT GCT ATC CTG		
Ad5 E1A-R1	GCG ACG CCC ACC AAC TCT CAC	HAdV5 E1A	This study
Ad5 E1A-F2	GAG CCT TGG GTC CGG TTT CTA TG		
Ad5 E1A-R2	CCA TTT TAG GAC GGC GGG TAG	HAdV5 E1A	This study
Ad5 hexon-F1	GAC GGA GCC AGC ATT AAG TTT GAT		
Ad5 hexon-R1	GTT GGC GGG TAT AGG GTA GAG CAT	HAdV5 hexon	This study
Ad5 fiber-F1	TAT TCA GCA TCA CCT CCT TTC C		
Ad5 fiber-R1	AAG CTA TGT GGT GGT GGG GC	HAdV5 fiber	This study
AdA1	GCT GAA GAA MCW GAA GAA AAT GA		
AdA2	CRT TTG GTC TAG GGT AAG CAC	Species A fiber	(20)
AdB1	TST ACC CYT ATG AAG ATG AAA GC		
AdB2	GGA TAA GCT GTA GTR CTK GGC AT	Species B fiber	(20)
AdC1	TAT TCA GCA TCA CCT CCT TTC C		
AdC2	AAG CTA TGT GGT GGT GGG GC	Species C fiber	(20)
AdD1	GAT GTC AAA TTC CTG GTC CAC		
AdD2	TAC CCG TGC TGG TGT AAA AAT C	Species D fiber	(20)
AdE1	TCC CTA CGA TGC AGA CAA CG		
AdE2	AGT GCC ATC TAT GCT ATC TCC	Species E fiber	(20)
AdF1	ACT TAA TGC TGA CAC GGG CAC		
AdF2	TAA TGT TTG TGT TAC TCC GCT C	Species F fiber	(20)

To determine whether >1 replication-competent serotype or strain was present in the samples with evidence of co-infection, we attempted to physically separate the paired co-infectants in sample 7151 by plaque purification. Of 92 plaques picked from the initial plate, all tested positive for HAdV-C by PCR and 12 of 92 also tested positive for HAdV-B. Several of the plaques that retained both HAdV-B and HAdV-C signals were replaqued, and PCR testing of these plaques yielded only HAdV-C isolates. Further efforts that used a detergent to increase separation within the original ITCF sample 7151 and applied the agarose overlay more quickly (6 hours) to prevent interplaque contamination also yielded only HAdV-C plaques (data not shown).

## Discussion

We demonstrate the rarely reported phenomenon of co-infections with multiple adenoviral species. Two previous studies have noted rare instances of HAdV-C dual infections in small numbers (27,28). HAdV-C, although rarely associated with pharyngitis outbreaks in recruits (10), is usually seen in children (7,8) and can produce latent infections that last into young adulthood. This fact, combined with low incidence of co-infection (27,28), has led to the assertion that multistrain adenovirus co-infections are not common (28) or clinically relevant. The results from the population tested in this study suggest otherwise. Samples from vaccinated recruits showed a high rate of co-infection with multiple species of adenovirus associated with adult ARD (HAdV-E and HAdV-B1).

Many of the identified co-infectants in this study were species not generally associated with ARD in the military (HAdV-C, HAdV-B2, and HAdV-F). Although these species were not likely the cause of ARD observed in these patients, since they are not believed to cause ARD in adults and because they have a high potential for latent carriage (1,7,8,29), their presence sheds new light on the general complexity of the human adenoviral load. In addition, they remain viable reservoirs capable of genetic complementation or recombination with upper respiratory strains.

Recombination can generate new strains with unique and stable phenotypes. Intraspecies adenovirus recombination has been demonstrated in laboratory cell-culture co-infection studies (30–32). These recombination events can generate viable hybrids with intermediate or unique immunogenic and tropic properties. Evidence suggests recombination can generate hybrids in immunocompromised patients (29,33,34), possibly as a result of co-infection with normally isolated serotypes. Recombination, particularly intraspecies, seems to play a major role in the evolution of new, virulent strains of HAdV (1,17,35,36). The currently dominant pathogenic HAdV in US military recruits, a considerably diverged variant HAdV-4 strain (16), appears to be a recent recombinant between HAdV-4 and a HAdV-B1 serotype, probably HAdV-7 (37). Given that these 2 are the most common co-infectants seen in our sample set, this finding suggests that the observed dominance of co-infections in vaccinated persons may have contributed to the emergence of the new variant. In general, the understanding and control of situations that create or promote co-infection may be important considerations.

The HAdV vaccine, an enteric-coated live-virus tablet designed to transiently and selectively infect the gastrointestinal tract with normal respiratory HAdV strains, contains viable HAdV-4 and HAdV-7. Thus, we cannot assume whether the detected co-infectants arose from the vaccine itself or from community acquisition of circulating strains. Most HAdV-4 strains in this study are not the vaccine strain but rather a highly divergent variant that has recently been dominant in military training centers (GenBank strain Z-G 95-873). This identity was shown by sequence analysis of 1,500 bp of the hexon gene from many primary infectants identified in the same sample set that was analyzed here (16). The variant HAdV-4 isolates consistently differ from the vaccine strain by 32 base substitutions, including 9 coding changes, in this region (16) (Table 1). Hexon sequence analysis showed that many HAdV-7 co-infectants are HAdV-7d2. HAdV-7d2 is distinguished from the HAdV-7 vaccine strain (HAdV-7a) by a single coding polymorphism in the hexon sequence, but this polymorphism (protein L443Q or nucleotide T1328A in GenBank [16]) is specific to HAdV-7d and HAdV-7d2 and is not found in HAdV-7a, b, c, g, or h or in the vaccine strain (16,38,39). Three of the other HAdV-7 co-infectants (1856, 4185, and 7372) were shown to be HAdV-7h by fiber gene sequencing. The fiber gene of HAdV-7h appears to have been horizontally transferred from HAdV-3 and thus is highly diverged from the usual HAdV-7 fiber gene, as found in the vaccine strain (17). Thus, sequence analyses show that most, if not all, co-infectants are currently circulating HAdV-4 and HAdV-7 strains that are distinct from the vaccine strains (16) (Table 1).

Four lines of evidence support the idea that most of the apparent genetic complexity in the throat swab samples comes from multiple strains, as opposed to recombinants with mixed genetic characteristics. The first comes from the microarray data. The microarray tests for hybridization of 6 independent probes designed to match serotype-specific sequences in 3 genes (18). Since different species do not cross-react among the microarray probes, hybridization of genes from 1 species to the identifying probes for 2 species would require redundant presence of 2 different alleles in all 3 genes. Since both natural recombination in hosts (17) and artificially encouraged recombination in cell culture (30,32) strongly favor homologous recombination and generation of nonredundant hybrid strains, redundant characterization of paired, divergent alleles is inconsistent with a single recombinant genome.

The second line of evidence supporting co-infection with independent genomes comes from comparisons of relative co-infectant titers before and after potentially selective events, such as growth in tissue culture. PCR amplification of fiber gene sequences using species B- and E-specific primers was performed on serial dilutions of vaccinated sample 7274 before and after passage of the original ITCF through 2 additional cycles of growth in A549 cells. In this instance, the relative titers of HAdV-4 and HAdV-7, as measured by serial-dilution PCR, changed by 2 orders of magnitude (data not shown). The rapid drift in relative concentrations of PCR targets from paired co-infecting strains strongly suggests that the co-infectants' genomes are replicating independently and thus likely to be physically separate entities.

The third line of evidence supporting co-infectant independence comes from whole-genome sequencing efforts. Several molecular methods indicated that vaccinated sample 7151 harbored an HAdV-5/HAdV-21 co-infection (Table 1 and Table 2). The genome of the HAdV-5 co-infecting strain was sequenced and assembled into a contiguous sequence (GenBank no. AY601635) consistent with a published HAdV-5 genome (GenBank accession no. AY339865) (40), which suggested no recombination of foreign DNA. However, this effort also generated several orphan sequences that did not fit into the assembled sequence and were subsequently identified as genetically redundant HAdV-21 regions. Further amplification and sequencing of several genetically distant fragments from the same sample using HAdV-21-specific primers yielded ≈2 kb of HAdV-21 sequence. On the basis of the entire genome and partial PCR sequencing analyses, >2 co-infecting HAdV genomes are contained in sample 7151.

The fourth line of evidence comes from our attempts to physically separate paired co-infectants by plaque purification. Sample 7151, which contained the HAdV-5/HAdV-21 co-infection, was used initially because it contained relatively equal titers of both co-infectants. Although most of the plaques tested contained HAdV-5, some contained both HAdV-5 and HAdV-21. Although we were unable to identify plaques that contained only HAdV-21, our results demonstrate the physical independence of the co-infecting entities and the functional independence of HAdV-5. Our results also suggest that either the HAdV-21 co-infectant is functionally dependent on HAdV-5 or is effectively outgrown by HAdV-5 to a degree that prevents independent isolation. Similar attempts were made with a few samples that had HAdV-4/HAdV-7 co-infections, but these were generally biased in titer (10^4^ in favor of HAdV-7) and, as expected, yielded only HAdV-7 in >300 plaques tested. The data demonstrated the functional independence of 1 co-infectant (HAdV-7) and physical independence of the co-infecting entities but could not conclusively demonstrate functional independence of the minor co-infectant.

Conventional clinical microbiologic methods, including microneutralization and hemagglutination inhibition, are comparative and designed to identify the primary HAdV serotype (or species) in a sample. Secondary infections are masked in these methods by the tests (e.g., microneutralization is reported as the strongest reaction, not the spectrum of reactions across all serotypes). Likewise, direct sequencing (16) may restrict identification to a single strain, particularly if 1 co-infectant is dominant. Restriction enzyme analysis methods are capable of resolving HAdV-C dual infections in which both serotypes are present in similar numbers (27). In contrast, when using sensitive molecular methods that can yield measurable signals from secondary (less numerous) co-infectants against the background of stronger signals produced by primary infecting strains, these methods may identify co-infections more than do conventional methods. In the case of respiratory infections, this finding has previously been documented (41).

Finally, each of the methods designed to test for multiple species or serotypes showed a higher number of HAdV (and accepted virulent HAdV species and serotypes) in vaccinated persons than in unvaccinated persons. HAdV vaccine was administered routinely to all trainees until supplies were exhausted, at which point adenovirus vaccination was stopped. Since trainees were vaccinated systematically, persons tended to be sampled at times when either all or no recruits were being vaccinated. Therefore, vaccinated samples collected and tested (from 1996 to 1998) are not concurrent with unvaccinated samples (collected from 1998 to 2000). Because of this sampling limitation, we could not confidently correlate HAdV co-infection with breakthrough infections in previously vaccinated persons. Thus, although this study highlights the previously underappreciated phenomenon of adenoviral co-infection, the conclusive examination of its relationship to vaccination must await reintroduction of HAdV vaccine (15).
